# Contribution of Body Mass Index Stratification for the Prediction of Maximal Oxygen Uptake

**DOI:** 10.7150/ijms.77818

**Published:** 2022-10-31

**Authors:** Fang Li, Cheng-Pang Yang, Cheng-You Wu, Chia-An Ho, Hung-Chih Yeh, Yuan-Shuo Chan, Wen-Sheng ChangChien, Chin-Shan Ho

**Affiliations:** 1 School of Physical Education, Central China Normal University, Wuhan, China.; 2 Department of Orthopedic Surgery, Division of Sports Medicine Chang Gung Memorial Hospital, College of Medicine, Chang Gung University, Linkou, Taiwan.; 3 Graduate Institute of Sports Science, National Taiwan Sport University, Taoyuan, Taiwan.; 4 Department of Special Education, National Taipei University of Education, Taipei, Taiwan.; 5 Innovation Lab., H2U Corporation, New Taipei, Taiwan.

**Keywords:** VO_2max_, 3-min incremental step-in-place, prediction model, BMI

## Abstract

The purpose of this study was to investigate whether modeling within separate body mass index (BMI) stratifications improves the accuracy of maximal oxygen uptake (VO_2max_) prediction compared to a model developed regardless of adults' BMIs. A total of 250 Taiwanese adults (total group, TOG) aged 22-64 years participated in this study, and were stratified into a normal group (NOG: 135), an overweight group (OVG: 69), and an obesity group (OBG: 46), according to the BMI classification recommended by the Taiwan Ministry of Health and Welfare. VO_2max_ was directly measured on an electromagnetic bicycle ergometer. Using the participant's heart rate in the 3-min incremental step-in-place test and demographic parameters, VO_2max_ prediction models established for four groups were TOG model, NOG model, OVG model, and OBG model, respectively. Compared with the TOG model, the OVG and OBG models had higher coefficients of determination and lower standard error of estimates (SEEs), or %SEEs. The validities of the NOG (r = 0.780), OVG (r = 0.776), and OBG (r = 0.791) models for BMI subgroups increased by 1.79%, 4.64%, and 8.22% respectively, and the reliabilities (NOG model: ICC = 0.755; OVG model: ICC = 0.765; OBG model: ICC = 0.779) increased by 3.18%, 3.27%, and 9.63%, respectively. These results suggested using separate models established in BMI stratifications can effectively improve the prediction of VO_2max_.

## Introduction

Obesity is a risk factor for various chronic diseases, including hypertension, cardiovascular disease (CVD), diabetes, and kidney disease 1-7, with CVD being the leading cause of death worldwide 8. Body mass index (BMI) is a standardized index calculated by dividing body weight (in kg) by height squared (in m^2^) and is used by the World Health Organization (WHO) to measure a person's degree of obesity: underweight, normal weight, overweight, and obese. BMI can be calculated easily and quickly and is therefore the most commonly used anthropometric indicator in research and clinical practice to assess obesity in the general population 4, 9, 10. Past studies have shown that overweight or obesity, described as higher BMI, is a major risk factor for cardiovascular disease in the general population 1, 2, 11. Reducing body weight to within the normal range has a positive effect on blood pressure and lipid levels, which are effective in reducing cardiovascular morbidity and all-cause mortality 12-15. The BMI thresholds for diagnosing obesity vary across different populations. Based on the association between various health conditions and BMI, WHO established, for European and North American populations, a normal BMI of 18.5-24.9 kg/m^2^; in contrast, a BMI of 25-29.9 kg/m^2^ is defined as overweight, and a BMI of 30 kg/m^2^ and above is defined as obese 3, 12, 16. However, using 30 kg/m^2^ as the BMI threshold for diagnosing obesity is too high for Asian populations and tends to underestimate health risks 17. Therefore, the Taiwan Ministry of Health and Welfare defines BMI of greater than or equal to 27 kg/m^2^ as obese, according to local population characteristics; A BMI between 24 kg/m^2^ and 27 kg/m^2^ is considered overweight, normal weight is defined as 18.5 ≤ BMI < 24 kg/m^2^, and BMI below 18.5 kg/m^2^ indicates underweight 17, 18.

Cardiorespiratory fitness (CRF) is an important indicator to assess cardiovascular health status in adults with different BMI levels 19, 20, and measuring CRF levels can predict the risk of future cardiovascular disease and all-cause mortality. Previous studies have shown a significant negative correlation between BMI and CRF in normal weight, overweight, and obese individuals, and adults with higher BMI levels typically have lower CRF levels 21, 22. The most direct and accurate measure of CRF is incremental cardiopulmonary exercise testing (CPET) on a treadmill or bicycle ergometer. In CPET, the plateau in VO_2_ reached by the participant at exhaustion represents the maximum upper limit of CRF 23. Therefore, maximal oxygen uptake (VO_2max_) is the best indicator of CRF levels in adults with various BMIs 24, 25. However, this approach has several drawbacks. Direct measurement of VO_2max_ requires expensive laboratory equipment, the participants must exercise until exhaustion, which is time-consuming, and maximum physical effort tends to increase the risk of adverse cardiovascular events in adults with higher BMI levels 20. Therefore, it is essential to explore low-risk and effective submaximal exercise solutions to indirectly measure VO_2max_ in adults with various BMIs.

Many scholars have proposed various submaximal exercise protocols in the past to predict VO_2max_ 26-29, and most of them developed VO_2max_ prediction formulas using age, sex, body mass, BMI, percent body fat (PBF), heart rate (HR), or distance to assess the CRF levels of adults with various BMIs using overall data. The most common field test is the 20-meter shuttle run test. It is simple, easy to administer, and convenient for simultaneous testing of multiple individuals 30. However, it requires a large space and is susceptible to environmental factors (rain, snow, etc.). To reduce the time and space costs of CRF testing, many studies have developed different step-up tests, such as the Young Men's Christian Association (YMCA), Queen's College, and Harvard Step tests, which require participants to continuously step onto and off a box of 20 to 50 cm height for three to five minutes 24, 31-34. However, in the most widely used 3-minute YMCA step test study, many scholars found that adults with higher BMIs were unable to complete the exercise test at standard intensity 35, 36, and they were prone to falling during the process of stepping onto and off the box. Therefore, an alternative to step-up tests, namely the 3 min incremental step-in-place (3MISP) test, has recently been proposed. Taking into account individual differences, the 3MISP test uses the midline between the middle of the anterior superior iliac spine and the patella as the target for knee elevation during stepping, without a step-up box, so it is safer and easier to complete than step-up tests. The prediction formula established by combining the exercise HR during the 3MISP test with demographic parameters can predict the VO_2max_ of healthy adults with relative accuracy 29, 37.

However, using the same prediction formula for adults with different BMIs may affect the accuracy of VO_2max_ estimation. Previous studies have found that the traditional approach to modeling VO_2max_ using overall data may overestimate VO_2max_ in individuals with low fitness levels and underestimate VO_2max_ in individuals with high fitness levels 28, 38-42. This overestimation or underestimation of VO_2max_ may be due to individual differences in participants, especially their degrees of obesity. To investigate whether modeling within separate BMI stratifications improves the accuracy of VO_2max_ prediction compared to a model developed regardless of adults' BMIs, this study stratified all participants (i.e., the total group, TOG) into three groups: the normal group (NOG), the overweight group (OVG), and the obesity group (OBG), according to the BMI classification criteria established by the Taiwan Ministry of Health and Welfare 17, 18. Then corresponding VO_2max_ prediction models were developed for each group. The effectiveness of the BMI stratified models was also compared with that of the VO_2max_ prediction model constructed using the TOG. In this study, it was hypothesized that the prediction models established within separate BMI stratifications (NOG, OVG, and OBG) would result in better VO_2max_ estimation than TOG model developed regardless of adults' BMIs.

## Materials and methods

### Study design

All participants (i.e., TOG) were stratified into three groups: NOG (18.5 ≤ BMI < 24 kg/m^2^), OVG (24 ≤ BMI < 27 kg/m^2^), and OBG (BMI ≥ 27 kg/m^2^), according to the BMI classification criteria established by the Taiwan Ministry of Health and Welfare 17, 18. Each participant completed the VO_2max_ and 3MISP tests. VO_2max_ was measured directly using an electromagnetic bicycle ergometer (Excalibur Sport Ergometer, Lode BV, the Netherlands). Chest strap heart rate sensors (Polar H10, Polar Electro Oy, Finland) were used to measure the heart rate response of participants during the VO_2max_ and 3MISP tests. VO_2max_ prediction models (i.e., the TOG, NOG, OVG, and OBG models, respectively) were developed for the TOG, NOG, OVG, and OBG by multivariate linear regression analysis. The validities and reliabilities of these prediction models were validated with the Pearson's correlation coefficient and intraclass correlation coefficient (ICC).

### Participants

A total of 250 healthy Taiwanese adults (124 males, 126 females) aged 22 to 64 years completed this study. None of the participants had medical histories of chronic diseases such as cardiovascular, skeletal or muscular diseases that might affect their ability to complete the exercise tests. The participants were divided according to the BMI classification criteria established by the Taiwan Ministry of Health and Welfare 17, 18, and the NOG, OVG, and OBG had 135, 69, and 46 participants, respectively. This study was approved by the Institutional Review Board of the Industrial Technology Research Institute (Hsinchu, Taiwan). All participants provided informed consent forms prior to participation in the experiment. And all experiments were conducted in accordance with relevant guidelines and regulations, i.e., the principles of the Declaration of Helsinki guidelines. In this study, the body weights and PBF of all participants were measured by body composition analyzer (InBody® 570, Biospace, Inc., Seoul, Korea), and BMI was calculated by dividing the participant's body weight (in kg) by the square of his/her height (in m^2^).

### Maximal graded exercise test

VO_2max_ was measured directly using the maximal graded exercise test (GXT) on a standard electromagnetic bicycle ergometer (Excalibur Sport Ergometer, Lode BV, the Netherlands). The initial workload was 25 W, followed by a progressive increase in resistance of 15 W every 2 minutes until the participant could no longer achieve the required pedaling frequency of 70 revolutions per minute 29. During the GXT, participants wore a chest strap heart rate sensor throughout the exercise to monitor their heart rate and used the Borg Rating of Perceived Exertion (RPE) Scale (6-20) to rate their exertion 43. Simultaneously, VO_2max_ was obtained and the respiratory exchange ratio (RER) of each participant was monitored with a cardiopulmonary exercise testing system (Vmax Encore 29 System, VIASYS Healthcare Inc., Yorba Linda, CA, USA). In this study, participants were considered to have achieved VO_2max_ if they met three of the following conditions: the participant's maximum heart rate reached more than ninety percent of the age-based maximum heart rate (220 - age); the RER was greater than or equal to 1.10; the increase in oxygen consumption began to plateau as the load continued to increase; and the RPE was greater than or equal to 18 28, 29.

### 3-min incremental step-in-place test

The 3MISP test began with a stepping frequency of 80 steps per minute (SPM) and then increased by 16 SPM every 30 seconds for 3 minutes. The heart rate response was recorded at the beginning of the exercise (HR0), at the first (HR1), second (HR2), and third (HR3) minutes into the exercise, and at the first minute after the end of the exercise (HR4). Participants were required to wear a heart rate sensor for monitoring of their heart rate response during the 3MISP test. The midpoint between the anterior superior iliac spine and the patella was measured and marked with colored tape as the height of knee elevation during stepping. Once the test began, the participant had to step to the tempo of a metronome, and each knee had to be raised to the indicated height. If the participant could not achieve the required knee height or keep up with the metronome for 30 seconds, then the 3MISP test was stopped and the data were excluded from the analysis 37.

### Statistical analysis

Multivariate analysis of variance was used to compare the differences in physical characteristics between the TOG, NOG, OVG and OBG, followed by the Bonferroni post-hoc test. The relationship between actual VO_2max_ measurements and other measurements in different BMI subgroups was evaluated, and the VO_2max_ predictive validity of the TOG, NOG, OVG and OBG models in each group was assessed by calculating the Pearson's correlation coefficients (r). Absolute r values between 0.00 and 0.10, between 0.10 and 0.39, between 0.40 and 0.69, between 0.70 and 0.89, and between 0.90 and 1.00 are indicative of negligible, weak, moderate, strong, and very strong correlations, respectively 44. Four VO_2max_ prediction models (i.e., the TOG, NOG, OVG, and OBG models) were developed by multiple stepwise regression analysis (training and verification sets were classified at 7:3 ratio), using the heart rate during the 3MISP test, age, sex (female = 0; male = 1), and body composition. The linearity, normality of residuals, and homoscedasticity assumptions of each model were checked using scatterplots, Shapiro-Wilk test/histograms of standardized residuals, and residual plots, respectively. We calculated variance inflation factor (VIF) to test the multi-collinearity of the datasets. Multivariate coefficients of determination (R²), standard error of estimate (SEE), %SEE, mean absolute error (MAE), and root mean squared error (RMSE) were used to analyze and compare the fit and accuracy of the TOG, NOG, OVG, and OBG models. Cross-validation analysis for each model was performed by the predicted residual error sum of squares (PRESS) statistical method 28, 29. The predictive reliability of these models for VO_2max_ in different BMI subgroups was validated by calculating ICCs (two-way mixed models; absolute agreement). For the ICC values, < 0.5 is regarded as poor, 0.5-0.75 as moderate, 0.75-0.9 as good, and > 0.90 as excellent reliability 45. Paired sample t-tests and Bland-Altman plots were used to compare the differences between the actual VO_2max_ measurements and the VO_2max_ estimates for each BMI subgroups 46. *p* less than 0.05 was considered to be statistically significant. All data in this study were analyzed in SPSS (version 22.0, IBM Corp., USA).

## Results

### The descriptive characteristics of the subjects

Table 1 presents the descriptive characteristics of the participants in the TOG, NOG, OVG, and OBG. The results of the multivariate analysis of variance showed that there were significant differences in BMI, PBF, and VO_2max_ among the TOG, NOG, OVG, and OBG (all *p* < 0.001). According to the post-hoc results, VO_2max_ values were higher in the TOG, NOG, and OVG than in the OBG by 4.10 (*p* = 0.002), 5.32 (*p* < 0.001), and 4.45 mL·kg^-1^·min^-1^ (*p* = 0.006), respectively.

### Correlation between the VO_2max_ and independent variables

Table 2 presents the Pearson's correlation coefficients between the actual VO_2max_ measurements and independent variables in the TOG, NOG, OVG, and OBG. The results showed that, in the TOG and NOG, age (TOG: r = -0.259, NOG: r = -0.270), PBF (TOG: r = -0.697, NOG: r = -0.712), and HR0 (TOG: r = -0.454, NOG: r = -0.501) were significantly negatively correlated with VO_2max_ (all *p* < 0.01). In addition, positive correlation was found between sex (female = 0, male = 1) and both ΔHR3-HR4 and VO_2max_ (TOG, sex: r = 0.461, ΔHR3-HR4: r = 0.573; NOG, sex: r = 0.542, ΔHR3-HR4: r = 0.543; all *p* < 0.01). In the OVG, there was negative correlation between age and VO_2max_ (r = -0.330, *p* = 0.006) but positive correlation between sex and both ΔHR3-HR4 and VO_2max_ (sex: r = 0.639, ΔHR3-HR4: r = 0.539, both *p* < 0.01). In the OBG, there was negative correlation between age (r = -0.294, *p* = 0.048), PBF (r = -0.760, *p* < 0.01), HR4 (r = -0.684, *p* < 0.01) and VO_2max_.

### Multivariate regression models for predicting VO_2max_

**Figure 1:**
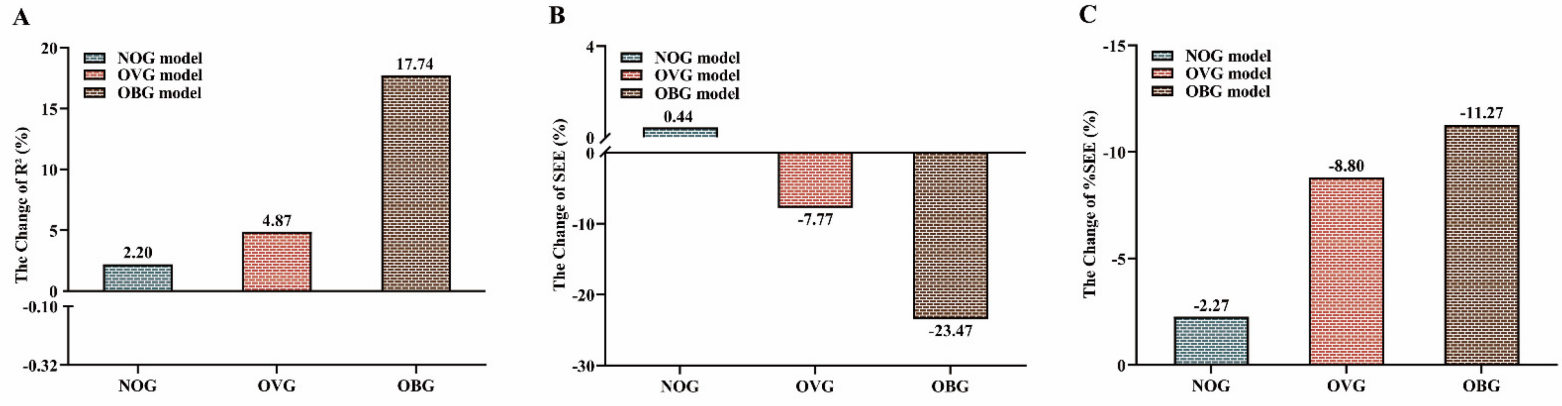
Percentage changes in R^2^ **(A)**, SEE **(B)**, and %SEE **(C)** of the NOG, OVG, and OBG models compared with the TOG model. NOG, normal group. OVG, overweight group. OBG, obesity group. SEE, standard error of estimate. %SEE, SEE/mean of measured VO_2max_ × 100.

Table 3 presents the multivariate regression models for predicting VO_2max_ in the TOG, NOG, OVG, and OBG. The VIFs for the TOG (1.036-2.642), NOG (1.101-2.019), OVG (1.017-1.112), and OBG (1.158-1.510) models were all less than 10 (Table 3), indicating that there was no multi-collinearity among the predictor parameters of each model 47. Figure 1 shows the percentage changes in R^2^ (Figure 1A), SEE (Figure 1B), and %SEE (Figure 1C) for the NOG, OVG, and OBG models developed within separate BMI stratifications compared with the TOG model including age, sex, PBF, BMI, HR0, and ∆HR3-HR4. The results showed that, compared with the TOG model (R^2^ = 0.637, SEE = 4.382 mL·kg^-1^·min^-1^, %SEE = 12.84%), the NOG model showed a 2.20% higher R² (0.651), a 0.44% higher SEE (4.401 mL·kg^-1^·min^-1^), and a 2.27% lower %SEE (12.55%); R² (0.668) was higher by 4.87%, SEE (4.041 mL·kg^-1^·min^-1^) was lower by 7.77%, and %SEE (11.71%) was lower by 8.80% for the OVG model; R² (0.750) was higher by 17.74%, SEE (3.353 mL·kg^-1^·min^-1^) was lower by 23.47%, and %SEE (11.39%) was lower by 11.27% for the OBG model. The cross-validation results of the PRESS method suggested that TOG, NOG, OVG, and OBG models had high cross-validities (∆R^2^: 0.01 to 0.014; ∆SEE: 0.043 to 0.193 mL·kg^-1^·min^-1^).

### Testing model assumptions

**Figure 2:**
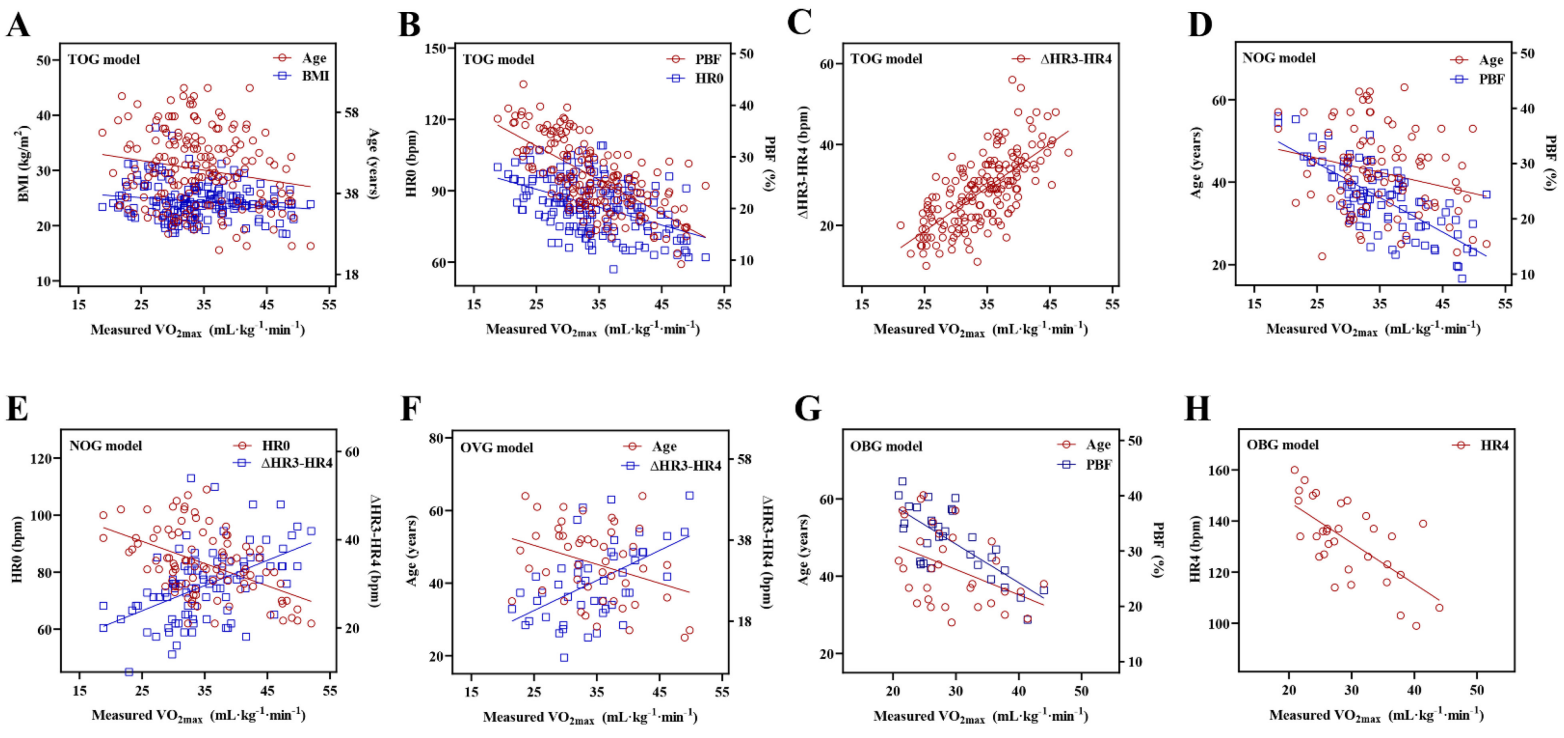
Scatter plots between the measured VO_2max_ and the independent variables within the TOG (A-C), NOG (D-E), OVG (F), and OBG (G-H) models.

**Figure 3:**
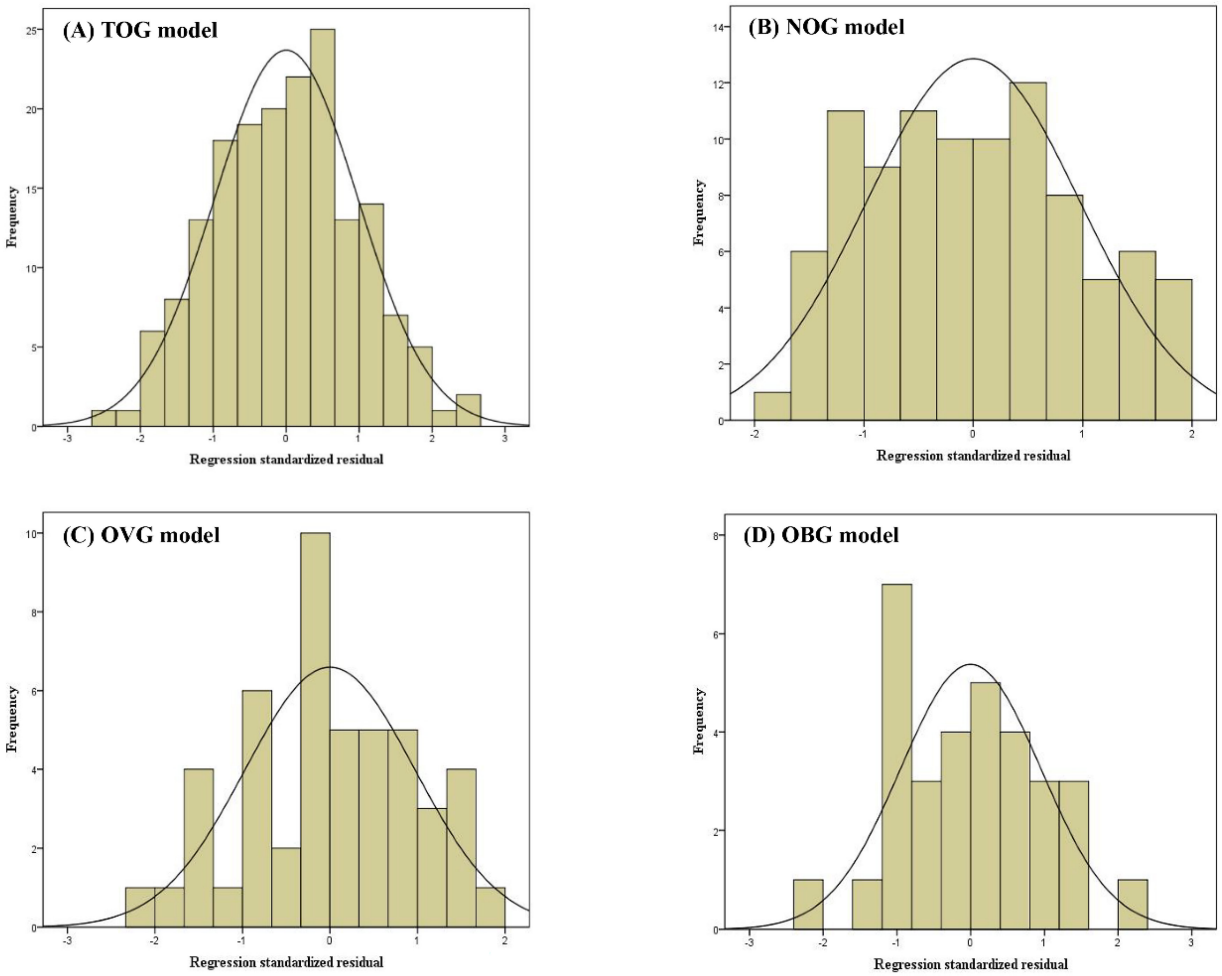
Histograms of standardized residuals for the TOG (A), NOG (B), OVG (C), and OBG (D) models.

**Figure 4:**
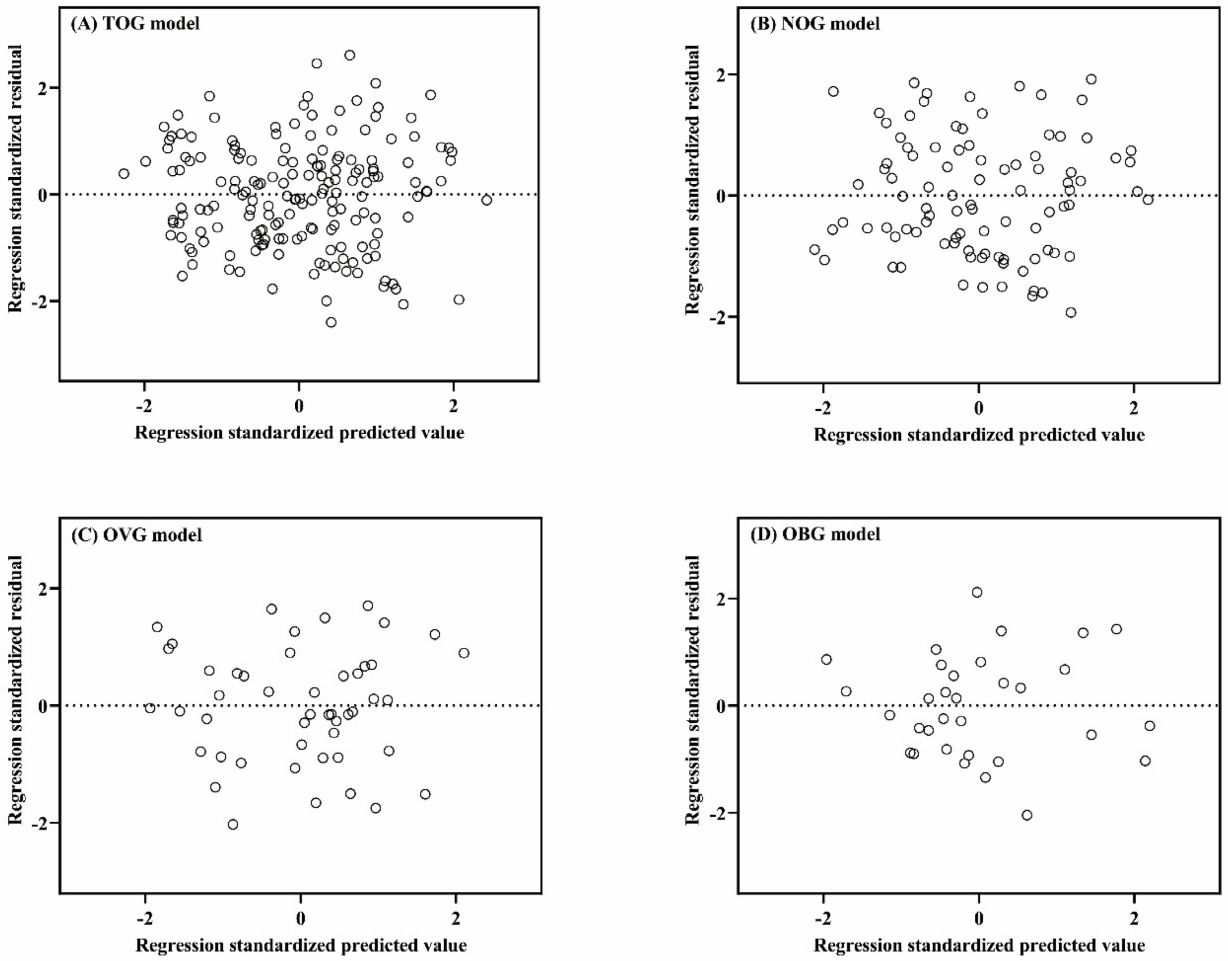
Scatter plots of the standardized residuals against regression standardized predicted value for the TOG (A), NOG (B), OVG (C), and OBG (D) models.

Linear regression assumptions (linearity, normality of residuals, and homoscedasticity) of TOG, NOG, OVG, and OBG models were all satisfied in this study. Figure 2 described the linear relationship between the measured VO_2max_ and the independent variables with the scatter plots. The results of the Shapiro-Wilk test indicated that the residuals within the TOG (*p* = 0.840), NOG (*p* = 0.055), OVG (*p* = 0.455), and OBG (*p* = 0.922) models were normally distributed. Histograms of the standardized residuals were also plotted to evaluate normality of residuals and to check whether there were outliers in each model (Figure 3). It could be found that standardized residuals of the TOG, NOG, OVG, and OBG models all followed normal distribution, and there were no outliers in their histograms. Homoscedasticity was tested using the scatter plots of the standardized residuals against regression standardized predicted value for each model. As shown in Figure 4, the residual plots of models were randomly scattered around the zero horizontal line, suggesting that the TOG, NOG, OVG, and OBG models all fulfilled the homoscedasticity assumption.

### Prediction accuracy of the regression model

The prediction accuracy of the TOG, NOG, OVG, and OBG models in the BMI subgroups was checked using performance metrics such as MAE and RMSE (Table 4). The MAEs and RMSEs of the TOG model (NOG: MAE = 3.79 mL·kg^-1^·min^-1^, RMSE = 4.53 mL·kg^-1^·min^-1^; OVG: MAE = 3.58 mL·kg^-1^·min^-1^, RMSE = 4.30 mL·kg^-1^·min^-1^; OBG: MAE = 3.32 mL·kg^-1^·min^-1^, RMSE = 3.99 mL·kg^-1^·min^-1^) for the BMI subgroups were all higher than those of NOG model (MAE: 3.72 mL·kg^-1^·min^-1^, RMSE: 4.44 mL·kg^-1^·min^-1^), OVG model (MAE: 3.16 mL·kg^-1^·min^-1^, RMSE: 3.98 mL·kg^-1^·min^-1^), and OBG model (MAE: 2.70 mL·kg^-1^·min^-1^, RMSE: 3.18 mL·kg^-1^·min^-1^). These results indicated that the regression models developed within separate BMI stratifications would result in better prediction accuracy than TOG model.

### Comparison between actual VO_2max_ measurements and VO_2max_ estimates

**Figure 5:**
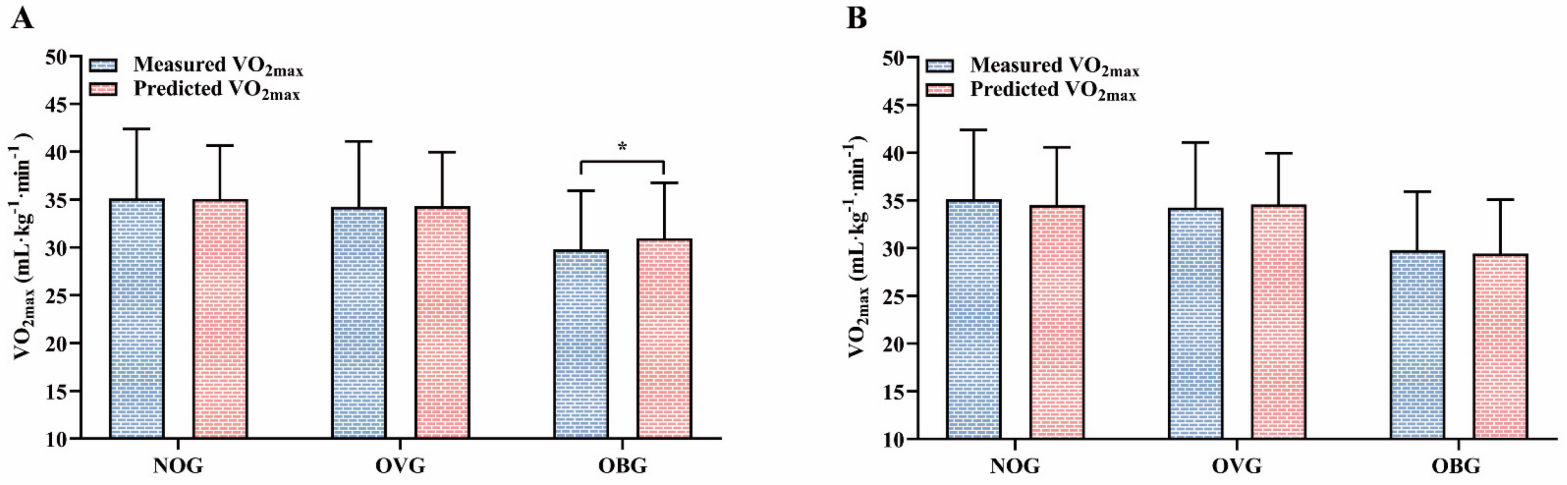
**(A)** Differences between the measured VO_2max_ and the VO_2max_ predicted by the TOG model in the NOG, OVG, and OBG. **(B)** Differences between the measured VO_2max_ and the VO_2max_ predicted by the NOG model, OVG model, and OBG model in the NOG, OVG, and OBG. NOG, normal group. OVG, overweight group. OBG, obesity group. * Significant difference between the measured and predicted VO_2max_ (*p* < 0.05).

Figure 5A presents the differences between actual VO_2max_ measurements and VO_2max_ estimates by the TOG model in the NOG, OVG, and OBG. Figure 5B shows the differences between the actual VO_2max_ measurements and the VO_2max_ values predicted by the NOG model, OVG model, and OBG model for different BMI subgroups. The results showed a significant difference between the measured VO_2max_ and the VO_2max_ predicted by the TOG model in the OBG (29.80 ± 6.12 mL·kg^-1^·min^-1^ vs. 30.96 ± 5.80 mL·kg^-1^·min^-1^, *p* = 0.049). In the NOG, OVG, and OBG, there were no statistically significant differences between the actual VO_2max_ measurements and the VO_2max_ values predicted by the NOG model, OVG model, and OBG model, respectively (NOG: 35.12 ± 7.26 mL·kg^-1^·min^-1^ vs. 34.52 ± 6.05 mL·kg^-1^·min^-1^; OVG: 34.25 ± 6.84 mL·kg^-1^·min^-1^ vs. 34.58 ± 5.38 mL·kg^-1^·min^-1^; OBG: 29.80 ± 6.12 mL·kg^-1^·min^-1^ vs. 29.42 ± 5.65 mL·kg^-1^·min^-1^; all *p* > 0.05).

### Validity and reliability of models for predicting VO_2max_

**Figure 6:**
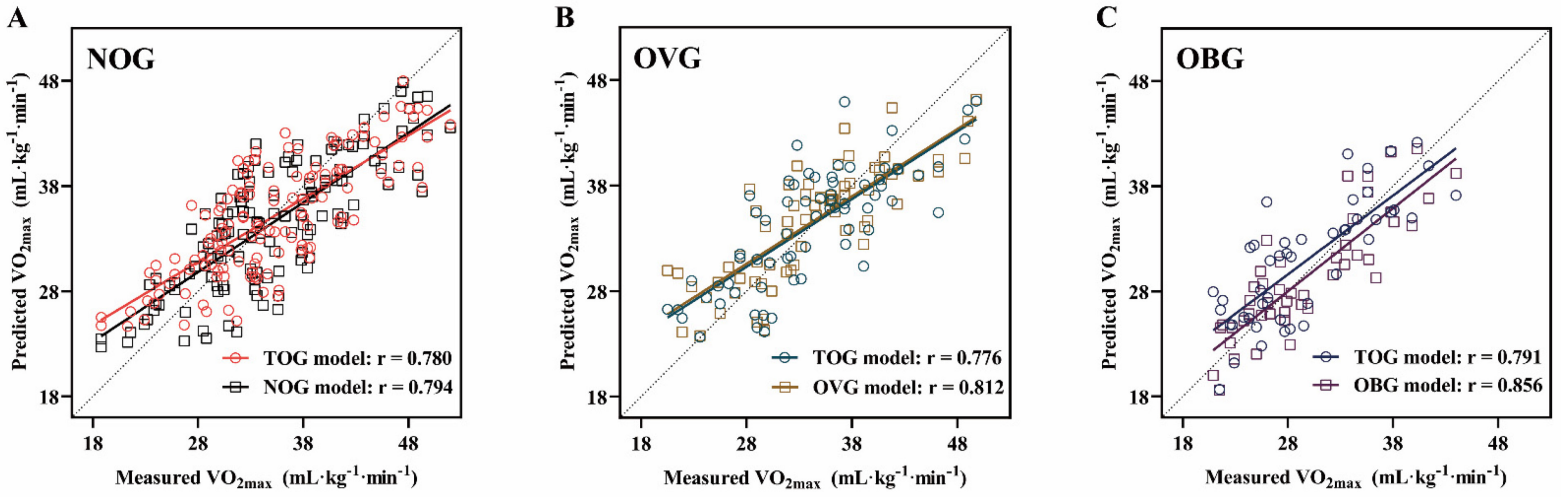
The relationships between the measured VO_2max_ and the VO_2max_ predicted by the TOG, NOG, OVG, and OBG models for the NOG** (A)**, OVG **(B)**, and OBG **(C)**. TOG, total group. NOG, normal group. OVG, overweight group. OBG, obesity group.

**Figure 7:**
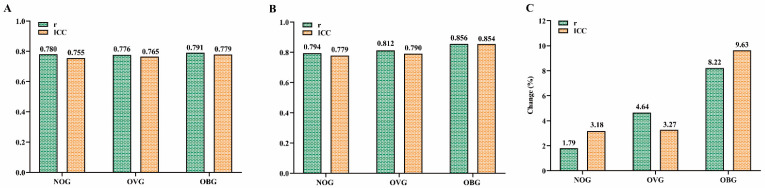
**(A)** The predictive validity (r) and reliability (ICC) of VO_2max_ in the TOG model for the NOG, OVG, and OBG. **(B)** The predictive validity (r) and reliability (ICC) of VO_2max_ in the NOG, OVG, and OBG models for the NOG, OVG, and OBG respectively. **(C)** Compared with the predictive validity (r) and reliability (ICC) of the TOG model for VO_2max_ in different BMI subgroups, the percentage changes in predictive validity (r) and reliability (ICC) of the NOG, OVG, and OBG models for VO_2max_ in each BMI subgroup. ICC, intraclass correlation coefficient. NOG, normal group. OVG, overweight group. OBG, obesity group.

Figure 6 presents the relationships between the actual VO_2max_ measurements in the NOG (Figure 6A), OVG (Figure 6B), and OBG (Figure 6C) and the VO_2max_ values predicted by the TOG, NOG, OVG, and OBG models, respectively. Figure 7A, B presents the validity analysis (r) and reliability analysis (ICC) of these four models for predicting VO_2max_ in different BMI subgroups. Figure 7B indicates that the NOG (r = 0.794, ICC = 0.779, both *p* < 0.001), OVG (r = 0.812, ICC = 0.790, both *p* < 0.001), and OBG (r = 0.856, ICC = 0.854, both *p* < 0.001) models had good validity and reliability in predicting VO_2max_ for each BMI subgroups^44,45^. Compared with the predictive validity and reliability of the TOG model for VO_2max_ in different BMI subgroups (NOG: r = 0.780, ICC = 0.755; OVG: r = 0.776, ICC = 0.765; OBG: r = 0.791, ICC = 0.779; all *p* < 0.001; Figure 7A), the NOG, OVG, and OBG models improved the predictive validities of VO_2max_ in the NOG, OVG, and OBG by 1.79%, 4.64%, and 8.22%, and the reliabilities by 3.18%, 3.27%, and 9.63%, respectively (Figure 7C).

### Bland-Altman analysis of VO_2max_ measured and predicted

**Figure 8:**
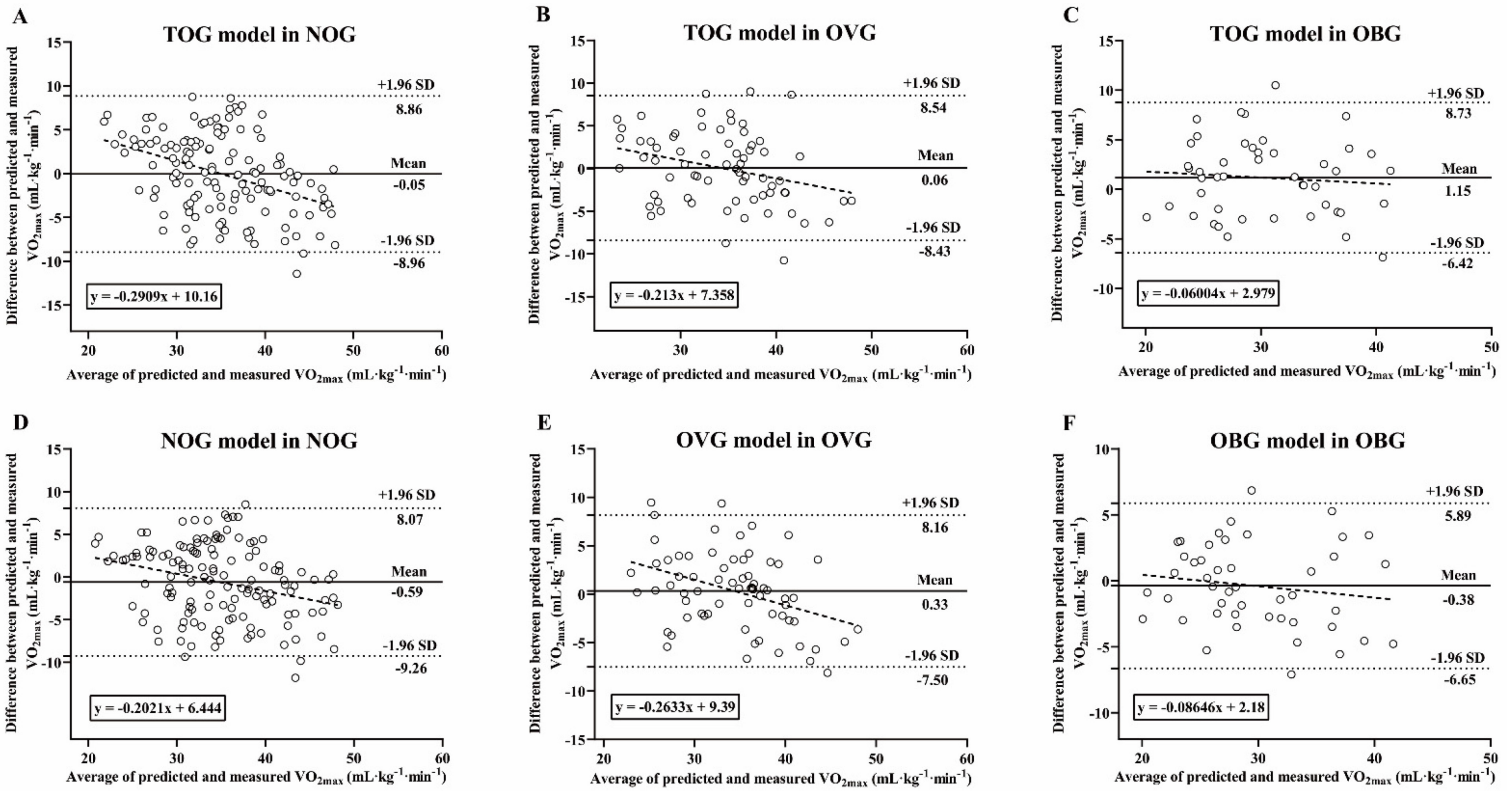
The differences between the predicted and measured VO_2max_ values were presented in Bland-Altman Plots, and the dotted line represents the regression line: **(A)** TOG model in NOG; **(B)** TOG model in OVG; **(C)** TOG model in OBG; **(D)** NOG model in NOG; **(E)** OVG model in OVG; **(F)** OBG model in OBG. TOG, total group. NOG, normal group. OVG, overweight group. OBG, obesity group.

Figure 8 presents Bland-Altman Plots including the linear regression between the difference and average of predicted and measured VO_2max_ in BMI subgroups. The results of Shapiro-Wilk test suggested that the residues were evenly distributed among the different VO_2max_ values in the NOG (TOG model: *p* = 0.148; NOG model: *p* = 0.17), OVG (TOG model: *p* = 0.966; OVG model: *p* = 0.652), and OBG (TOG model: *p* = 0.672; OBG model: *p* = 0.645). The mean difference between the VO_2max_ values predicted by the TOG model and the actual VO_2max_ measurement values in the NOG and OVG were -0.05 mL·kg^-1^·min^-1^ (*p* = 0.893) and 0.06 mL·kg^-1^·min^-1^ (*p* = 0.911), respectively, and the 95% limits of agreement (LoA) were -8.96 to 8.86 mL·kg^-1^·min^-1^ and -8.43 to 8.54 mL·kg^-1^·min^-1^, respectively (Figure 8A, B). In the OBG, there was a significant difference between the VO_2max_ values predicted by the TOG model and the actual VO_2max_ measurements (mean differences = 1.15 mL·kg^-1^·min^-1^, *p* = 0.049), with a 95% LoA of -6.42 to 8.73 mL·kg^-1^·min^-1^ (Figure 8C). There were no significant differences between the actual VO_2max_ measurements and those predicted respectively by the NOG, OVG, and OBG models in each BMI subgroup (all mean differences from -0.59 to 0.33 mL·kg^-1^·min^-1^, *p* > 0.05), and the corresponding % LoA in the NOG, OVG, and OBG were -9.26 to 8.07 mL·kg^-1^·min^-1^, -7.50 to 8.16 mL·kg^-1^·min^-1^, and -6.65 to 5.89 mL·kg^-1^·min^-1^, respectively (Figure 8D-F).

## Discussion

In the past, many studies have used the overall data from adults with various BMIs to establish a VO_2max_ prediction formula with a considerable degree of reliability and validity, and they also supported the application of submaximal exercise to assess CRF 24, 25, 29, 48, 49. However, overestimation or underestimation of VO_2max_ by the prediction formula has been found in some studies based on submaximal exercise. This phenomenon may be due to individual differences, especially in specific groups, such as those with high or low levels of physical fitness 28, 37, 42. However, few studies have further investigated this phenomenon. Further investigation based on key factors is particularly important for analyzing the causal relationship between it and the predictivity of VO_2max_. The WHO recommends the use of BMI classification to assess the degree of obesity in the general population, overweight or obesity increases the risk of cardiovascular disease 1, 11, 12. The correlation between BMI and CRF is significantly negative, and adults with higher BMI usually have lower CRF levels 21, 22.

Therefore, in this study, the TOG was stratified into three groups (i.e., NOG: 18.5 ≤ BMI < 24 kg/m^2^, OVG: 24 ≤ BMI < 27 kg/m^2^, OBG: BMI ≥ 27 kg/m^2^) according to the BMI classification criteria established by the Taiwan Ministry of Health and Welfare 17, 18, and corresponding VO_2max_ prediction models (i.e., the NOG, OVG, and OBG models) were developed for each BMI subgroup and compared in terms of validity and reliability with the TOG model. The results of this study supported our original hypothesis, modeling after stratification by BMI increased R^2^ and decreased %SEEs for the prediction of VO_2max_ in the NOG, OVG and OBG. In addition, this study also demonstrated that establishing separate prediction models within BMI stratifications can further improve the predictive validity and reliability of VO_2max_ for each BMI subgroup, as well as the agreement between the measured and predicted VO_2max_. The accuracy of VO_2max_ prediction will be affected if the same prediction model is used for adults with various BMIs. Therefore, using separate prediction models developed within BMI stratifications is recommended for VO_2max_ estimation. Members of the general public can use the corresponding VO_2max_ prediction model to assess their own CRF levels with reference to the appropriate BMI subgroups (i.e., NOG, OVG, or OBG), which can provide a basis for the development or adjustment of later exercise programs.

The models for predicting VO_2max_ in the TOG, NOG, OVG, and OBG were developed by the multiple stepwise regression analysis. Eventually, the independent variables selected for the TOG model were age, sex, PBF, BMI, HR0, and ∆HR3-HR4; The independent variables selected for the NOG model were age, sex, PBF, HR0, and ∆HR3-HR4; The independent variables selected for the OVG model were age, sex, and ∆HR3-HR4; The independent variables selected for the OBG model were age, PBF, and HR4. Each of the independent variables (i.e., age, sex, PBF, BMI, and 3MISP-HR) used in this study was significantly related from VO_2max_ (Table 2), which is consistent with previous studies indicating that age, sex, physical characteristics (PBF or BMI), and HR are important predictors of VO_2max_ 28, 29, 31, 37, 41, 51. In particular, heart rate is a physiological indicator of cardiac and circulatory system function. Previous studies have shown a linear relationship between exercise heart rate and VO_2max_ during the 3MISP test 29, 37, and the results of this study supported this view. In this study, HR0 and HR4 during the 3MISP test were negatively correlated with VO_2max_, and ∆HR3-HR4 was positively correlated with VO_2max_ in the NOG, OVG and OBG, as well as in the TOG (Table 2). Studies by Matsuo et al. 28 and Chung et al. 37 also reported that heart rate at the beginning of, during, and after exercise were significantly and negatively correlated with VO_2max_, and the decrease in heart rate after exercise was positively correlated with VO_2max_. Clearly, heart rate is an important factor in predicting VO_2max_. By continuously monitoring the heart rate response during the 3MISP test, we can objectively understand the load on the participant's body during exercise 19, and improve the accuracy of VO_2max_ prediction models in different BMI subgroups.

The results of this study indicated that the TOG model including age, sex, PBF, BMI, and 3MISP-HR (i.e., HR0, ∆HR3-HR4) overestimated VO_2max_ in the OBG (Figure 5A), which is consistent with previous studies reporting that the VO_2max_ prediction formula using the overall data will overestimate VO_2max_ in individuals with low fitness levels and underestimate it in individuals with high fitness levels 28, 38-42. This overestimation of VO_2max_ in individuals with low fitness levels may increase the risk of adverse cardiovascular events. To improve the accuracy of VO_2max_ estimation and reduce the estimation error, in this study, all subjects were stratified into three groups (i.e., NOG, OVG, and OBG) according to the BMI classification criteria established by the Taiwan Ministry of Health and Welfare, and corresponding VO_2max_ estimation models (i.e., NOG, OVG, and OBG models) were developed for each BMI subgroup. The results of this study showed that the explained amount (R^2^) of VO_2max_ in the NOG, OVG, and OBG models increased by 2.20-17.74%, SEE changed by 0.44-23.47%, and %SEE decreased by 2.27-11.27% (Figure 1) as compared with the TOG model, and their MAEs and RMSEs were all lower (Table 4) in BMI groups. The predicted values of VO_2max_ in the NOG, OVG, and OBG models were not significantly different from the actual VO_2max_ measurements of each BMI subgroup (Figure 5B). These results imply significant differences in CRF levels among adults with different BMIs (Table 1), which may affect the accuracy of VO_2max_ prediction if the same prediction model is used. In contrast, developing separate prediction models within BMI stratifications can effectively improve the predictivity of VO_2max_ and reduce the error.

To further evaluate the validities and reliabilities of the VO_2max_ prediction models based on BMI subgroups, this study employed the Pearson's correlation coefficient and ICC statistical methods for the NOG, OVG, and OBG models 44, 45 and compared the predictive validities and reliabilities of VO_2max_ in the NOG, OVG, and OBG with the TOG model constructed using the TOG. The results of this study showed that the validities of NOG, OVG, and OBG models increased by 1.79-8.22%, and the reliabilities increased by 3.18-9.63% comparing to the TOG model for BMI subgroups (Figure 7). In previous studies, many scholars have developed feasible VO_2max_ prediction models regardless of an individual's BMI. They also found that these prediction models overestimated VO_2max_ in individuals with low fitness levels and underestimated VO_2max_ in those with high fitness levels 38-40, 42. The results of this study indicated that developing separate VO_2max_ prediction model within BMI stratifications can significantly improve the predictive validity and reliability of VO_2max_ in adults with various BMIs.

The Bland-Altman plot is one of the most suitable statistical methods for assessing the agreement between two quantitative measures 46, 50, and many previous studies have applied this method to analyze the agreement between direct and indirect measures (i.e., VO_2max_ prediction models) of VO_2max_ 28, 29, 37, 52, with considerable success. Therefore, in this study, Bland-Altman analysis was used to evaluate and compare the agreement between the methods for predicting VO_2max_ in the NOG, OVG, and OBG with the TOG model and direct measurement of VO_2max_, as well as the agreement between establishing separate VO_2max_ prediction models (i.e., the NOG, OVG, and OBG models) within BMI stratifications and direct VO_2max_ measurement. The results of this study showed that the 95% LoAs between the VO_2max_ values predicted by the TOG model and the actual VO_2max_ measurements in the NOG, OVG and OBG were larger than those of the VO_2max_ prediction models developed within separate BMI stratifications (i.e., the NOG, OVG, and OBG models) for each BMI subgroup (Figure 8). Moreover, in OBG, the mean difference between the actual measured VO_2max_ values and those predicted by the TOG model was significant (1.15 mL·kg^-1^·min^-1^, *p* = 0.049; Figure 8C), while no significant differences were found between the actual measured VO_2max_ values and those predicted by the OBG model (Figure 8F). These results implied higher agreement between the method of predicting VO_2max_ for each BMI subgroup by developing BMI stratified models and the direct VO_2max_ measurement method than that of a model established regardless of adults' BMIs. Therefore, to improve the accuracy of VO_2max_ prediction, it is recommended that corresponding prediction models be developed within separate BMI stratifications for predicting VO_2max_ in adults with various BMI levels.

In summary, the BMI stratification approach for VO_2max_ prediction proposed in this study achieved good results, and similar approaches need to be further explored, especially when applied to other demographics, such as older adults and patients. This will help to improve the accuracy of CRF assessment and practical application in fitness/rehabilitation.

## Limitations and suggestions

There are certain limitations in this study. First, our subjects are healthy adults aged 20-64 years, so we cannot know the stability of using the model in this study to predict VO_2max_ in children, adolescents, elders, or individuals with diseases. Second, the BMI stratification in this study is carried out according to the BMI classification criteria established by the Taiwan Ministry of Health and Welfare, thus the stratification models may not be suitable for other racial groups. Future research should increase the diversity of samples to verify the applicability of our prediction models to the wider population. Finally, this study is a cross-sectional rather than a longitudinal study, so causal inference cannot be made. Further follow-up studies are needed in the future.

## Conclusions

In this study, we have developed relatively accurate prediction models for estimating VO_2max_ in healthy adults with various BMIs, and the general public can use the corresponding VO_2max_ prediction model to assess their CRF levels with reference to their BMI classification subgroup (i.e., NOG, OVG, or OBG), which can provide a basis for the development or adjustment of their exercise training programs. The traditional approach of building a VO_2max_ prediction model regardless of an individual's BMI, i.e., using the same prediction formula for adults with different BMIs, will affect the accuracy of VO_2max_ estimation. Establishing separate VO_2max_ prediction models within BMI stratifications can further reduce the SEE or %SEE values of BMI subgroups, improving both the predictive validity and the reliability, as well as the agreement between the measured and predicted VO_2max_. These results indicated that BMI can be regarded as a basis for the stratification, and it is recommended to use BMI stratified models for VO_2max_ prediction.

## Figures and Tables

**Table 1:** The descriptive characteristics of the subjects.

	TOG (N = 250)	NOG (N = 135)	OVG (N = 69)	OBG (N = 46)	p	Range
Age (years)	43.3 ± 10.0	42.8 ± 10.1	45.6 ± 10.2	41.5 ± 9.2	0.132	22.0-64.0
Male (N)	124	49	43	32		
Female (N)	126	86	26	14		
Height (cm)	166.1 ± 8.2^d^	164.2 ± 8.2^d^	167.2 ± 7.5	170.0 ± 7.4^ab^	<0.001	150.0-188.0
Body mass (kg)	67.4 ± 12.9^bd^	59.5 ± 8.2^acd^	70.8 ± 7.3^bd^	85.4 ± 10.1^abc^	<0.001	43.5-123.9
BMI (kg/m^2^)	24.2 ± 3.3^bcd^	21.9 ± 1.7^acd^	25.3 ± 0.9^abd^	29.5 ± 2.4^abc^	<0.001	18.5-37.8
PBF (%)	26.2 ± 7.0^d^	24.3 ± 6.3^d^	26.3 ± 6.8^d^	31.5 ± 6.4^abc^	<0.001	9.2-44.1
VO_2max_ (mL·kg^-1^·min^-1^)	33.9 ± 7.2^d^	35.1 ± 7.3^d^	34.2 ± 6.8^d^	29.8 ± 6.1^abc^	<0.001	18.8-52.0
HR0 (bpm)	83 ± 11	83 ± 12	82 ± 11	86 ± 11	0.212	57-109
HR4 (bpm)	129 ± 17	129 ± 17	127 ± 19	133 ± 16	0.242	83-161
ΔHR3-HR4 (bpm)	28 ± 9	29 ± 9	28 ± 10	25 ± 7	0.056	9-56

TOG, total group. NOG, normal group. OVG, overweight group. OBG, obesity group. PBF, percent body fat. BMI, body mass index. HR0, heart rate at the start of the 3MISP test. HR4, heart rate at the first minute after the 3MISP test. ΔHR3-HR4, the difference in heart rate between the third minute into the 3MISP test and the first minute after the test. Values are presented as the mean ± standard deviation. ^a^ Significantly different from the TOG, *p* < 0.05. ^b^ Significantly different from the NOG, *p* < 0.05. ^c^ Significantly different from the OVG, *p* < 0.05. ^d^ Significantly different from the OBG.

**Table 2:** Pearson's correlation coefficients between VO_2max_ and independent variables in each group.

Groups	Variables	VO_2max_	Age	Sex	PBF	HR0	HR4
TOG	Age (years)	-0.259**					
	Sex (female = 0, male = 1)	0.461**	0.001				
	PBF (%)	-0.697**	0.174**	-0.457**			
	HR0 (bpm)	-0.454**	-0.004	-0.184**	0.414**		
	HR4 (bpm)	-0.452**	-0.092	-0.262**	0.407**	0.631**	
	ΔHR3-HR4 (bpm)	0.573**	-0.180**	0.200**	-0.411**	-0.451**	-0.616**
NOG	Age (years)	-0.270**					
	Sex (female = 0, male = 1)	0.542**	-0.006				
	PBF (%)	-0.712**	0.246**	-0.593**			
	HR0 (bpm)	-0.501**	0.074	-0.192*	0.451**		
	HR4 (bpm)	-0.427**	0.023	-0.262**	0.407**	0.613**	
	ΔHR3-HR4 (bpm)	0.543**	-0.196*	0.264**	-0.419**	-0.457**	-0.597**
OVG	Age (years)	-0.330**					
	Sex (female = 0, male = 1)	0.639**	-0.025				
	PBF (%)	-0.537**	0.129	-0.732**			
	HR0 (bpm)	-0.308**	-0.145	-0.170	0.308**		
	HR4 (bpm)	-0.352**	-0.219	-0.220	0.317**	0.721**	
	ΔHR3-HR4 (bpm)	0.539**	-0.179	0.164	-0.216	-0.398**	-0.629**
OBG	Age (years)	-0.294*					
	Sex (female = 0, male = 1)	0.554**	-0.004				
	PBF (%)	-0.760**	0.234	-0.557**			
	HR0 (bpm)	-0.470**	0.067	-0.299*	0.468**		
	HR4 (bpm)	-0.684**	-0.134	-0.511**	0.551**	0.507**	
	ΔHR3-HR4 (bpm)	0.655**	-0.221	0.435**	-0.596**	-0.515**	-0.633**

TOG, total group. NOG, normal group. OVG, overweight group. OBG, obesity group. PBF, percent body fat. HR0, heart rate at the start of the 3MISP test. HR4, heart rate at first minute after the 3MISP test. ΔHR3-HR4, the difference in heart rate between the third minute into the 3MISP test and the first minute after the test. * *p* < 0.05; ** *p* < 0.01.

**Table 3:** Multiple regression models predicting VO_2max_ (mL·kg^-1^·min^-1^) in the TOG, NOG, OVG, and OBG.

Models	Variables	B	β	*p*	VIF	R^2^	SEE	%SEE	R^2^*p*	SEE*p*
TOG model	Constant	52.991		<0.001		0.637	4.382	12.84	0.623	4.550
	Age (years)	-0.092	-0.123	0.010	1.036					
	Sex (female = 0, male = 1)	5.213	0.366	<0.001	2.222					
	PBF (%)	-0.300	-0.288	<0.001	2.642					
	BMI (kg/m^2^)	-0.352	-0.160	0.017	2.066					
	HR0 (bpm)	-0.085	-0.135	0.018	1.488					
	∆HR3-HR4 (bpm)	0.213	0.264	<0.001	1.406					
NOG model	Constant	53.695		<0.001		0.651	4.401	12.55	0.637	4.444
	Age (years)	-0.093	-0.132	0.050	1.101					
	Sex (female = 0, male = 1)	3.668	0.250	0.002	1.532					
	PBF (%)	-0.447	-0.385	<0.001	2.019					
	HR0 (bpm)	-0.131	-0.208	0.006	1.401					
	∆HR3-HR4 (bpm)	0.178	0.201	0.006	1.310					
OVG model	Constant	29.888		<0.001		0.668	4.041	11.71	0.661	4.234
	Age (years)	-0.167	-0.260	0.005	1.017					
	Sex (female = 0, male = 1)	7.640	0.551	<0.001	1.112					
	∆HR3-HR4 (bpm)	0.268	0.371	<0.001	1.094					
OBG model	Constant	77.740		<0.001		0.750	3.353	11.39	0.740	3.291
	Age (years)	-0.208	-0.318	0.004	1.158					
	PBF (%)	-0.426	-0.405	0.002	1.510					
	HR4 (bpm)	-0.197	-0.477	<0.001	1.368					

PBF, percent body fat. BMI, body mass index. HR0, heart rate at the start of the 3MISP test. HR4, heart rate at first minute after the 3MISP test. ΔHR3-HR4, the difference in heart rate between the third minute into the 3MISP test and the first minute after the test. B, unstandardized regression weights. β, standardized regression weights. SEE, standard error of estimate. %SEE, SEE/mean of measured VO_2max_ × 100. R^2^*p*, PRESS squared multiple correlation coefficient; SEE*p*, PRESS standard error of estimate.

**Table 4:** Prediction accuracy of the regression model in the NOG, OVG, and OBG.

	NOG		OVG		OBG	
	TOG model	NOG model	TOG model	OVG model	TOG model	OBG model
MAE	3.79	3.72	3.58	3.16	3.32	2.70
RMSE	4.53	4.44	4.30	3.98	3.99	3.18

MAE, mean absolute error. RMSE, root mean squared error.

## Abbreviations

VO_2max_: maximal oxygen uptake
BMI: body mass index
TOG: total group
NOG: normal group
OVG: overweight group
OBG: obesity group
SEE: standard error of estimate
CVD: cardiovascular disease
WHO: World Health Organization
CRF: Cardiorespiratory fitness
CPET: cardiopulmonary exercise testing
PBF: percent body fat
HR: heart rate
YMCA: Young Men's Christian Association
3MISP: 3 min incremental step-in-place
HR0: heart rate at the start of the 3MISP test
HR4: heart rate at first minute after the 3MISP test
ΔHR3-HR4: the difference in heart rate between the third minute into the 3MISP test and the first minute after the test
ICC: intraclass correlation coefficient
GXT: graded exercise test
RPE: rating of perceived exertion
RER: respiratory exchange ratio
SPM: steps per minute
